# Who should pay for the continuity of post-trial health care treatments?

**DOI:** 10.1186/s12939-019-0919-0

**Published:** 2019-06-03

**Authors:** Roberto Iunes, Manuela Villar Uribe, Janet Bonilla Torres, Marina Morgado Garcia, Juliana Alvares-Teodoro, Francisco de Assis Acurcio, Augusto Afonso Guerra Junior

**Affiliations:** 1 The World Bank, 1818 H Street, NW, Washington, DC 20433 USA; 2 Unión Europe to communications of the ACTÚE Colombia Project, Brussels, Belgium; 3 Department of Social Pharmacy, Federal University of Minas Gerais, Av. Presidente Antônio Carlos, 6627 – Pampulha, Belo Horizonte, Minas Gerais 31270-901 Brazil

**Keywords:** Equity, Treatment of patients, Post-trial access

## Abstract

**Background:**

The bioethical debate in the world on who should pay for the continuity of post-trials treatment of patients that have medical indication remains obscure and introduces uncertainties to the patients involved in the trials. The continuity of post-trial treatment was only incorporated in the 2000s by the Helsinki Declaration. The Universal Declaration on Bioethics and Human Rights, published in 2006, points out that post-trial continuity may present a broader scope than just the availability of the investigated medicine. In the latest version of this Declaration, in 2013, it was stated that “prior to the start of the clinical trial, funders, researchers and governments of the countries participating in the research should provide post-trial access for all participants who still require an intervention that was identified as beneficial. This information should also be disclosed to participants during the informed consent process”. However, a systematic review on the registration of phase III and IV clinical trials, from the clinical trials website, demonstrated that the understanding of the various guidelines and resolutions is conflicting, generating edges in the post-trial setting. For the health authorities of countries where clinical trials take place, the uncertainties about the continuity of the treatments generate gaps in care and legal proceedings against health systems, which are forced to pay for the treatments, even if they are not included in the list of medicines available to the population.

**Methods:**

Fifty-one representatives from the health, judicial, legislative, patient and academic organizations of eight countries of Latin American and South Korea took part in a meeting in Chile, in 2017, to discuss the responsibility of the treatment continuation after clinical trials. From a hypothetical case of development of a new drug and its studies of efficacy and safety, the participants, divided in groups, proposed recommendations for the problem and pointed out the pros and cons of adopting each recommendation. The groups were, afterwards, confronted by a simulated jury and, finally, issued a final recommendation for the problem. Then, an analysis was made on the content of the recommendations and the pros and cons in adopting conservative or liberal positions, besides the possible impacts of a restrictive regulation regarding the conduction of clinical trials, pointed out by the groups, before and after the simulated jury.

**Results:**

The theme was widely discussed and about 12 recommendations were proposed by the participants. The main ones took into account aspects related to patients’ rights, economic factors and the development of new technologies, above the position of industry and research institutes, as well as the legislation in force in each country.

**Conclusion:**

The countries of Latin America and South Korea, currently, do not have laws that address patients’ rights, moreover, there is no definition on who should be responsible for post-trial treatments. It is suggested that the World Health Organization issue a resolution recommending that all associated countries determine that the pharmaceutical and medical device industries, or those that sponsored it, should continue to provide treatment to all patients who participated in clinical trials and have medical indication to the continuity.

## Background

The search for new molecules in the past years has evidenced new conflicts about the participation of human beings in clinical investigations. Since the end of World War II, where research was conducted with humans in concentration camps until the last revision of the Helsinki Declaration, much has evolved in terms of ethical issues in human research and the rights of patients involved in clinical trials [6]. However, the bioethical debate in the world on who should shoulder the continuity of post-clinical trials for patients with medical indication remains obscure and introduces uncertainties to the patients involved in the trials. In this sense, continuity of treatment after the end of the clinical trial has been ethically debated around the world since 1980 among researchers, health professionals, drug regulatory agencies, ethics committees, and research volunteers [5]. A study focus on staff experiences of closing out a clinical trial involving withdrawal of treatment revealed that the ending of a clinical trial may be challenging. As the trial progressed, patients became increasingly anxious about withdrawal of treatment. Staff not only had to take funding pressures and patient distress into account, but they also found themselves caught between an ethic of Hippocratic individualism and one of utilitarianism [9].

The post-clinical treatment continuity was only incorporated in the 2000s by the Declaration of Helsinki [5]. In 2003, the International AIDS Society (IAS) defined continuity of treatment as: the ethical responsibility to compensate volunteer individuals who agreed to participate in the research and who were exposed to risks, invasive procedures, among others [8].

The Universal Declaration on Bioethics and Human Rights, published in 2006, points out that post-research continuity may present a broader scope than just the availability of the investigated drug [10]. In the latest version of this Declaration, in 2013, it was stated that “prior to the start of the clinical trial, funders, researchers and governments of the countries participating in the research should provide post-survey access for all participants who still require intervention identified as beneficial. In addition, this information should also be disseminated to participants during the informed consent process [17]”. The International Ethical Guidelines for Biomedical Research Involving Human Subjects created by The Council for International Organizations of Medical Sciences (CIOMS/WHO) [15] is the main international ethical document about post-trial access to medicines and is very clear when attributing to the researcher and the sponsor the responsibility of supplying the drug and monitoring the participants in the cases when the medication is beneficial.

The first studies about the post-trial have emerged in the context of patients with HIV/AIDS. Among them, researches with members of Research Ethics Committees (REC), researchers and patients involved in research in several countries (43% were from Latin America), except the United States of America, that answered that the drugs should be provided to all infected people in the world if the benefits were proven [11]. Since then many stakeholders have discussed the subject, however, post-trial access still is a controversial topic in the literature. The legislation and guidelines are inconsistent and ambiguous and fail to provide clear information on the situations in which access must be guaranteed, for how long, and who is responsible for the provision [12, 14].

In a study conducted with the opinion of 93 individuals who participated in clinical trials, the researchers pointed out that post-research continuity may exceed the availability of the investigational drug, being its broader reach [13], conclusions similar to the Declaration Universal Declaration on Bioethics and Human Rights, signed by 191 countries [15].

A systematic review (SR) of 312 clinical trials conducted between 2004 and 2007, involving patients with HIV/AIDS, malaria and tuberculosis, showed that only four studies mentioned the continuity of post-research treatment [3], what shows how the post-research scenario has many edges. Another SR published in 2009, in the clinical trials’ registry of phase III and IV, showed that the understanding of the application of the different guidelines and resolutions is conflicting, since of the 31 protocols included in the study: 14 (45%) focuses on women, 12 (39%) reported on the drug (10 offered the medication investigated); and five (16%) reported some health care. In addition, studies that provided the investigational drug did so differently (eight sponsored by the industry, six for all participants up to commercial availability or for a defined period, and two for patients who completed the experimental arm) [1].

The reasons why drug trial participants should, or need not be ensured post-trial access to the medicines was investigated in a SR and the authors concluded that there is a wide variety of reasons justifying post-trial access to drugs, many of them biased. The SR alerts to the need for caution in the use of sub-sets of literature, especially when used for decision-making. It also emphasizes that research on ethics, costs, viability and legality of the post-trial access is necessary [14].

In this context, the purpose of this manuscript is to describe the discussions around the post-trial access of patients involved in clinical research by a group of Latin American stakeholders involved in decision-making in the fields of law and health in their countries.

## Methods

Multi-Stakeholder engagement approaches have been used to ensure participation on a specific issue. They aim to ensure participatory equity, accountability and transparency, as well as the creation of partnerships and networks amongst different stakeholders for improved dialogue and decision-making in all stages of planning and implementation [16]. In this way, support the use of a multi-stakeholder approach in policy design can represent an important contribution to a successful policy implementation when discussing the potential pros and cons of this implementation. It can also be an important avenue for action to support the equitable realization of the right to health in health systems such as those in Latin America.

The paper describes the discussions held at the Sixth Meeting of the SaluDerecho Initiative of the World Bank and the Chilean Ministry of Health, in September 2017 in Santiago, Chile, whose main theme was “Ethics and transparency in access to medicines.” The objective of the meeting was to strengthen the transdisciplinary approach between rights and health systems. In addition, it sought to open an opportunity to discuss challenges and new approaches to health and law. It also provided a space for the dissemination and exchange of actions and experiences of countries seeking to improve the health rights and health systems efficiency situation, regarding medicines and other health technologies.

The 51 participants were representatives of health, judicial, legislative, academic, and patient organizations from various Latin American countries: Brazil, Chile, Costa Rica, Colombia, Ecuador, Mexico, Uruguay and South Korea. Everyone involved actively participate and have reference positions in their countries, which allows them to interfere in the decision-making process in the health and law fields.

The participants were divided into small groups and provoked, by means of a hypothetical case, to answer the following question: “Who should pay for the continuity of post-trial health care treatments?”

The hypothetical case concerned the conduct of a clinical trial to test a new drug, a monoclonal antibody, for the treatment of metastatic breast cancer in which, at the end of the clinical trial, it was concluded that 30% of the patients would benefit from a further 6 months of use.

After reading the case, the participants received the following guidelines for the discussion:

- A consensus is not necessary. All points of view are valid.
- The debate and the exchange of experiences aim to contribute to the decision-making process of the various actors involved in the issue: judges, managers, academics, among others, from the health systems of the countries involved.
- This activity aims to contribute to a better understanding of the challenges and implications in the field of health and the law in the continuous access to medicines and other health technologies for individuals who undergo clinical research.

After the discussions the participants, should answer the following questions:

- In cases where breast cancer patients require more than six cycles of drug application, who should bear the costs of ongoing treatment (family, researcher, or government)? Keep in mind that the treatment has not yet been evaluated or incorporated by the health system.
- What are the pros and cons of adopting regulations that require research centers (universities, institutes/NGOs, and businesses) to maintain the continued provision of treatment after the end of clinical research?

The recommendations suggested by the participants, pros and cons, were grouped according to the responsibility of bearing the costs of ongoing treatment: the family, the sponsor, the researcher and the government. The qualitative analysis of the collected data was done adopting procedures of the content analysis. Initially, the information was coded, whereby raw data are systematically transformed and grouped into units that allow the description of relevant characteristics of their content. Thus, the main ideas cited there were extracted or inferred from the textual data, categorizing their content. Finally, the main results were described and discussed [7].

## Results

The central question “Who should pay for the continuity of post-trial health care treatments?” was discussed and some proposals are described in Tables 1, 2 and 3. The recommendations, pros and cons, were listed taking into account aspects related to patients’ rights, economic factors and the development of new technologies, besides the position of industry and research institutes, as well as the legislation in force in each country.

**Table 1:** Pros and cons of making patients and their families responsible

Patients and Families	
Pros	Cons
Reduce the final cost of medicines	Could limit the access of low-income persons / families
Make the conduction of clinical trials more attractive to sponsors	
Can make patients and families more compliant to treatment

**Table 2:** Pros and cons of making sponsors responsible

Sponsors	
Pros	Cons
Avoid family expenses	Could weakens the local clinical research groups participating in the trials and decreases research
Protects the patient’s health	Could jeopardizes the financing of the tests
Immediate access to medication for the patient / Ensuring continuity of treatment (gives patient peace of mind - possibility of placebo effect)	Reduce industry investment in countries with high prevalence of rare diseases
The public health system would not pay	Could create an iatrogenic risk for the patient to whom the medication is given even without registration if there is no ongoing medical follow-up.
The cost to the industry would be defined more clearly, knowing the future (the cost is internalized, knowing that it will have to be paid)	Could make the treatment more expensive and unfeasible

**Table 3:** Pros and cons of making researchers and the state responsible

Researches and State	
Pros	Cons
Inclusion of clinical trials in public health policy	Inconsistent with official procedures to cover drugs with proven evidence
Prioritize disease research	Could make the cost of clinical trials in the country prohibitive.
State could impose conditions for the study	May require change in legislation
Guaranteed completion of the study	
State could request / obtain best price	
Greater transparency	

Empowering patients and their families by continuing treatment was the option considered the least interesting. According to the participants, considering the family as responsible or co-responsible implies in hurting established ethical precepts and would be a setback in the achievements already reached in terms of patients’ rights. Another possibility is to making sponsors responsible for the continuity of treatments when necessary (Table 2).

The recommendation to make sponsors responsible for the continuity of the treatments had many good points, but also important negative points, especially as regards the chance of clinical research being impaired in the countries. Participants weighed the recommendation and some alternatives to minimize possible negative points were raised. An alternative is to oblige sponsors to provide the drugs while they are not evaluated and incorporated into health systems. Another alternative would be to establish a fund for maintenance of the treatments (including all necessary care) to ensure continuity, to treat possible adverse events and to improve the quality of care for post-trial patients. Besides these, another alternative to reduce the possible economic losses of the companies is the reduction of the tax rates or the granting of other incentives to encourage the researches in the countries.

The possibility of holding the States accountable for the continuity of the treatments, before registration and approval for commercialization, according to the group of participants, can be a complicated and sensible alternative, since the countries have in their legislations criteria for the incorporation and availability based on results of efficacy, safety and cost-effectiveness of medicines. Engaging states in research funding could jeopardize countries’ budgets, with resources already limited to guaranteeing access to medicines.

Another recommendation is the creation of a fund for shared responsibility between sponsors, researchers and the state, in order to guarantee the continuity of treatment for patients, without hindering clinical research in the countries. Co-accountability can help in rationalizing risks, with all parties aware of their responsibilities the patients would be safer. One caveat to this recommendation, raised by the participants, is the possibility of using this fund for other proposals, thereby compromising the continuity of treatments. On the other hand, a reserve can be created to support future investigations in cases where all the resources are not used.

Another important recommendation is about the necessity of regulamentation of biomedical research, preferably by law, because it is related to fundamental rights, in each country before any research is undertaken. The regulation should guarantee the participant’s interests and rights broadly, with clear obligations of industry and universities, institutes, hospitals, researchers participating in the research.

As a final recommendation, the group proposed that the World Health Organization (WHO) draft a resolution recommending all associated countries to oblige the medicine and medical device industries or those who have sponsored it to continue to provide treatment. The treatment should be provided to all patients who have participated in clinical trials, who have an indication for the drug, regardless their legal situation (drug approval/registration) or inclusion in the list of medicines in the health system of that country.

## Discussion

This is a current and relevant topic, but extremely delicate because it involves different stakeholders and points of view. It is a fact that the issue of post-trial access for drugs touches on a much broader discussion, especially about the participation of developing countries in international clinical research. This concern is related to the possibility of participation of these countries only to avoid more rigorous ethical supervision or the use of economically disadvantaged research subjects in order to accelerate recruitment, for example, without guarantee of the supply of the medicine after the end of the study, if its benefits have been proven [4].

Silva et al. [12] analysed clinical trials registered on EU Clinical Trials Register (EUCTR) and noted that in high-income countries (HIC), 54% of the clinical trials lacked plans for post-trial access. In the low (LMIC) and upper middle-income countries (UMIC), only 38% of trials lacked plans for post-trial access. In the HIC, 55% of clinical trials lacking plans for post-trial access involved vulnerable populations. In LMIC and UMIC, 71 and 76% of clinical trials without plans for post-trial access, respectively, involved vulnerable populations. So, it is important to note that post-trial care safeguards participants who do not have or have insufficient access to health care outside of research. These participants include people from low-income and middle-income countries and people who are uninsured or otherwise lack sufficient access to health care in high-income countries [2].

Undoubtedly, the continuity of the research drug supply after the conclusion of the study is mandatory when there is benefit to the patient and he/she does not present a therapeutic alternative. Thus, it becomes unethical to discontinue treatment in cases where there is evidence of efficacy. For Dainesi [4], it is still a situation of necessity and not only of benefit to the volunteers of the research. Among the countries involved in the discussions, only Brazil and Chile have specific laws that require continuity of treatment after the end of clinical trials. In Brazil, the clinical trial sponsor must guarantee the treatment for an unspecified period, or while the patient is benefiting from the treatment. In Chile, the institution that obtained authorization to conduct the clinical trial in the country, or the holder of the product registration, is responsible for providing the treatment and the provision must take place while the patient is receiving clinical benefits [12].

The recommendation proposed by the participants of the meeting corroborates the results found in a study conducted by Dainesi [4] in which the author also describes that there was a consensus on the part of the research groups, members of the REC and also sponsors that, with continuity of treatment, this should be provided by the sponsor and free of charge. However, although widely discussed, obligations to post-clinical patients refer only to medical care, not covering all the care they need [2]. In addition, there are many recommendations for cases where medicines bring benefits to patients, but lack the necessary care regulations when medicines fail in their primary goals, in special in order to maintain access to continued monitoring, treatment for complications, or existing treatment alternatives [14].

This paper presents some limitations. One of them is the fact that, despite the participation of multi-stakeholders, representatives of the pharmaceutical industries or professionals directly involved in clinical trials were not part of the group. Such condition may have influenced the construction of the final recommendation. However, in spite of the limitation, it is the position of a group of professionals directly involved with the decision-making in the health issues of their countries and demonstrates how representatives of the judiciary, managers and academics face this question.

## Conclusion

Many countries of Latin America and South Korea do not have, to date, laws that ad dress patients’ rights and there is no definition of who should be responsible for post-trial access to treatments. In addition, a large part of the population of that country can be considered as vulnerable, which increases the need for regulation of research. The multi-stakeholders discussion on the subject brings up possibilities or possible paths to be followed in order to ensure that patients are assisted after the research is completed. One way of achieving this, according to the participants, could be the World Health Organization (WHO) draft a resolution recommending all associated countries to oblige the industries or those who have sponsored the clinical trial to continue to provide treatment to all patients who have an indication for the drug.

## Abbreviations

CIOMS: International Organizations of Medical Sciences
EUCTR: EU Clinical Trials Register
HIC: High-income countries
HIV/AIDS: Human Immunodeficiency Virus/ Acquired Immunodeficiency Syndrome
REC: Research Ethics Committees
SR: Systematic Review
WHO: World Health Organization
